# An Experimental Model of Meningoencephalomyelitis by Rocio Flavivirus in Balb/C Mice: Inflammatory Response, Cytokine Production, and Histopathology

**DOI:** 10.4269/ajtmh.2011.10-0246

**Published:** 2011-08-01

**Authors:** Veridiana Ester Dias de Barros, Fabiano P. Saggioro, Luciano Neder, Rafael Freitas de Oliveira França, Viviane Mariguela, Juliana Helena Chávez, Sandra Penharvel, Jorge Forjaz, Benedito Antônio Lopes da Fonseca, Luiz Tadeu Moraes Figueiredo

**Affiliations:** Virology Research Center, Department of Pathology, Department of Surgery and Anatomy of the School of Medicine of Ribeirão Preto, University of São Paulo, Brazil

## Abstract

Rocio virus (ROCV) is a flavivirus, probably transmitted by *Culex* mosquitoes and maintained in nature as a zoonosis of wild birds. Rocio virus caused a human epidemic of severe encephalitis that lasted from 1973 to 1980 in the Ribeira valley, in the southeastern coast of Brazil. After this outbreak, serologic evidence of ROCV circulation has been reported and public health authorities are concerned about a return of ROCV outbreaks in Brazil. We show here a study on the pathogenesis and the physiopathology of ROCV disease in the central nervous system of a Balb/C young adult mice experimental model. The animals were intraperitoneally infected by ROCV and followed from 0 to 9 days after infection, when all of them died. Nervous tissue samples were collected from infected animals for immunohistochemistry and molecular biology analysis. We observed the virus in the central nervous system, the inflammatory changes induced by Th1 and Th2 cytokines, and the final irreversible damage of nervous tissues by neuronal degeneration and apoptosis. These findings can help to better understand the pathogenesis and physiopathology of the human meningoencephalomyelitis by ROCV and other flaviviruses.

## Introduction

Members of the genus *Flavivirus* (*Flaviviridae*) are positive-sense, single-stranded RNA viruses that infect humans and other vertebrates. These viruses are mainly transmitted by bites of hematophagous arthropods (mostly mosquitoes and ticks). Currently, in this genus, more than 60 distinct viruses have been recognized worldwide with several others being classified as either tentative species or genotypes of other viruses.^1^ The genomic RNA consists of a unique open reading frame (ORF) that is flanked by non-coding regions that, together, form a secondary stem-loop structure required for RNA translation, replication, and/or expression including pathogenetic determinants.^2^ The flavivirus ORF encodes 10 proteins including three structural (capsid [C], pre-membrane [prM], and envelope [E])] and seven non-structural (NS1, NS2a, NS2b, NS3, NS4a, NS4b, and NS5) proteins.^3^ Based on nucleotide sequences, phylogenetic trees separate flavivirus in tick-borne and mosquito-borne branches. The mosquito-borne branch includes two major clades, the *Culex*-borne and the *Aedes*-borne.^4^ Rocio virus (ROCV) is included in the *Culex*-borne clade and is closely related to Ilhéus virus (ILHV). The ROCV is the first flavivirus uniquely indigenous of Brazil that has the genome completely sequenced and characterized.^5^ Other viruses in the *Culex*-borne cluster are Saint Louis encephalitis virus (SLEV), Japanese encephalitis virus (JEV), Murray Valley virus (MVV), and West Nile virus (WNV) all highly important for public health worldwide.

Although both ROCV and ILHV are neurotropic and together share a subclade in the phylogenetic tree, unlike the members of the neurotropic JEV group of viruses transmitted by *Culex* mosquitoes, both viruses have been most often isolated from *Psorophora* mosquitoes.^6^–^8^ However, ROCV can infect *Culex* mosquitoes under laboratory conditions.^9^

An outbreak of ROCV encephalitis occurred in the southeastern region of Brazil during 1973–1979 affecting at least 23 counties at Ribeira Valley, in the southern coast of São Paulo State. The ROCV disease was characterized by an acute onset of fever and headache that evolved to seizures and/or alteration of consciousness, resulting in a case/fatality ratio of 10%. The most frequent central nervous system (CNS) symptoms were meningeal irritation (57.3%), alteration of consciousness (51%), and motor abnormalities (49.65%), especially gait and impaired equilibrium. Blindness and deafness were also reported.^10^–^12^ Furthermore, about 20% of the disease survivors developed sequelae such as motor abnormalities (49.65%), especially gait and impaired equilibrium.^13^ The important aspects of ROCV epidemiology remain unknown and causes for virus appearance and disappearance in the Ribeira Valley remain unknown. The ROCV was isolated from the brain of a fatal encephalitis patient and also from a wild bird, *Zenothrichia capensis.* Serologic studies during the outbreak suggested that wild birds could be virus reservoirs. The ROCV was also isolated from the mosquito *Psorophora ferox*^14^ and, in 2004, two birds captured in the south of Brazil presented ROCV antibodies.^15^ Thus, ROCV remains a threaten public health problem.

We show here a study on the pathogenesis and the physiopathology of the meningoencephalomyelitis by ROCV in a mouse experimental model, analyzing aspects of the histopathology including inflammatory response and cytokine production, looking for the virus, and also for messenger RNA (mRNA) of cytokines in nervous tissues.

## Materials and Methods

### Animals.

Young adult Balb/C mice were obtained from the Animal Facility and maintained in isolated cages at the Virology Research Center, both in the School of Medicine of the University of São Paulo in Ribeirão Preto, Brazil. The experiments were approved by the Ethical Committee on Vertebrate Animal Experiments of the University of São Paulo (no. 080/2004).

### Virus.

The ROCV SPH34675 strain (at passage 3) used for mouse infection was kindly supplied by Dr. Terezinha Lisieux Coimbra, Adolpho Lutz Institute, São Paulo State, Brazil. Virus stocks were obtained from brains of intracerebrally infected suckling mice. Mouse brains in phosphate buffered saline (PBS) (dilution 1:20 wt/vol) were macerated and centrifuged at 2,000 × *g* for 10 min at 4°C and supernatants were stored at −70°C as virus stock.

### Infection of mice by ROCV and negative controls.

Fifty, 4-week-old females, of wild-type young adult Balb/C mice, were infected intraperitoneally (IP) with 10,000 LD_50_ of ROCV in a volume of 200 μL/mouse. This large virus dose produced encephalitis and death in 100% of the mice. The animals were monitored daily and most of them developed encephalitis signs such as hind limb paralysis, tremors, muscle weakness, ruffled pile, and difficulty to feed. The animals were killed on Days 0, 2, 4, 6, 7, 8, and 9 after infection (a.i.). The ROCV reached the CNS rapidly after inoculation, because its genome was found in the brain at the same day, 2 h a.i. (Day 0). A negative control mouse group (*N* = 30) and a sham-infected mouse group were also included in the experiments. Negative control mice (*N* = 30) were inoculated IP with PBS pH 7.4 and sham-infected mice (*N* = 14) were inoculated IP with supernatants of macerated uninfected mouse brains. Negative control mice and sham-infected mice were also killed on Days 0, 2, 4, 6, 7, 8, and 9 a.i. and their nervous tissues analysis was used for comparison with those of ROCV-infected animals.

### Animal sacrifice and nervous tissue collection.

Before sacrifice, the animals were anesthetized by inhalation with ether and were exsanguinated by cardiac puncture. The chest cavity was quickly opened and 15 to 20 mL of a 0.9% NaCl solution followed by a 10% neutral buffered formalin solution were injected directly into the left ventricle to perfuse the central nervous tissue. After fixation, the body was dissected and samples of entire brain and spinal cord were removed and placed into 10% neutral buffered formalin solution for 24 h, at room temperature (RT), to allow proper fixation. For experiments using real-time polymerase chain reaction (PCR), mice were anesthetized as previously described and perfused with 0.9% NaCl solution. Samples of the entire brain and spinal cord were removed and triturated in PBS using a tissue Power Gen 125 grinder (Fisher Scientific, AL, USA). These samples were stored at −70°C until use.

### Tissue preparations.

Tissue samples were dehydrated in an ethanol gradient (50%, 70%, 80%, and 100%), embedded in paraffin (Paraplast, Merck, USA), and submitted to serial sections (3 μm in thickness) in a rotary microtome (Reichert Jung, Heidelberg, Germany). Tissue sections were mounted on silane precoated glass slides (Sigma, St. Louis, MO). Histological assessment was carried out following routine hematoxylin and eosin staining and the sections were examined by light microscopy.

For immunohistochemistry analysis, all tissue sections were deparaffinized by three washes in xylene and rehydrated in an ethanol gradient (100%, 80%, 70%, and 50%). The tissue sections were immersed in a 3% H_2_O_2_ solution for 30 min, to block endogenous peroxidase activity and submitted to an antigen retrieval heating step following the protocol recommended by the manufacturer (see Table 1). To eliminate nonspecific binding sites, the tissue sections were blocked with a solution including 1 part of rat or mouse (depending on the secondary antibody used in the test) serum and 6 parts of PBS. The incubation period of 2 h, at RT, was followed by another incubation, at RT, overnight, with the primary antibodies (Table 1) diluted in a solution of 1.5% bovine serum albumin in PBS. After washing in 0.05% Tween 20–Tris-buffer saline solution, the tissue sections were incubated with the biotinylated secondary antibodies, for 60 min, and it was followed by addition of an avidin-biotinylated horseradish peroxidase complex and diaminobenzidine (Novocastra, Newcastle upon Tyne, UK), as recommended by the manufacturer. Tissue sections were then quickly counterstained with Meyer's hematoxylin. For negative controls, primary antibodies were omitted of the staining process.

Specifically stained cells (endothelial cells, neurons, astrocytes, and macrophage-type cells) were identified and quantified under a light microscope. At least 20 areas of 0.0625 mm^2^ were randomly selected for analysis under high magnification (×400), and the number of positive cells per square millimeter was determined. The same amount was examined for determination of tumor necrosis factor α (TNF-α)-producing cells The score used was 1+ = < 10% positive cells; 2+ = 10–50% positive cells; 3+ = 50–90% positive cells; 4+ = > 90–100% positive cells.

### RNA extraction, reverse transcription, PCR, and nested PCR for ROCV detection.

The RNA was extracted from the brain and spinal cord of the animals by Trizol reagent, following the protocol recommended by the manufacturer (Invitrogen- Life Technologies, CA, USA). The RNA extracts from the brain and spinal cord, collected at different days a.i., were submitted to a reverse transcription (RT)-PCR for detection of ROCV using *Flavivirus* genus-specific primers (Flavivirus-S: TCA AGG AAC TTC ACA CAT GAG ATG TAC T) and Flavivirus-C: GTG TCC CAT CCT GCT GTG TCA TCA GCA TAC A) followed by a ROCV species-specific nested PCR, using primers Flavivirus-S (TCA AGG AAC TTC ACA CAT GAG ATG TAC T) and ROCV-C (TCA CTC TTC AGC CTT TCG).^16^ The RNA extract from the virus stock was used as positive control and uninfected mice brain and sham-infected mice collected 9 days a.i. were used as negative controls.

For the ROCV-specific RT-PCR, 1 μg of each RNA sample was submitted to reverse transcription by using SuperScript III enzyme (Invitrogen- Life Technologies, CA, USA), random primers. The mixture was incubated at 50°C, for 1 h and at 70°C, for 15 min. Next, the RT product was submitted to a PCR in a reaction mixture of 50 μL including, 200 mM of Tris-HCl (pH 8.4), 500 mM of KCl, 50 mM of MgCl_2_, 10 mM of each one of the deoxynucleoside triphosphates (dNTPs), 1 U/μL of Platinum Taq DNA polymerase (Invitrogen-Life Technologies), 15 mM of Flavivirus-S and Flavivirus-C primers (Table 2) and 5 μL of RT products. The PCR mixture was submitted to 30 cycles of amplification at 94^°^C for 1 min, at 53^°^C for 1min, at 72^°^C for 1 min, and finally 72^°^C for 5 min. Five microliters of the products of this first reaction were used for a nested PCR containing the same reaction mixture described previously, except for the inclusion of 15 mM of Flavivirus-S and ROCV-C primers (Table 2). The mixture was submitted to 25 cycles at 94°C for 1 min, at 53°C for 1min, 72°C for 2 min, and finally, 72°C for 5 min. Amplicons obtained in the nested PCR were visualized under UV light after electrophoresis in 2% agarose gel and ethidium bromide staining.

### Quantitative analysis of cytokine mRNA by real-time PCR.

Cytokine mRNA of extracts from mouse nervous tissues, collected at different days a.i., were quantified by real-time RT-PCR using specific commercial kits (Bionner, CA, USA). The tests were done using the SybrGreen PCR MasterMix (Applied Biosystems, Warrington, UK) in an ABI Prism 7000 Sequence Detection System (Applied Biosystems). Primer sequences, predicted amplicon sizes, annealing, and melting temperatures, are shown in Table 3; 10 μM of each specific primer pair and 5 μL of complementary DNA (cDNA) were used in each reaction. The standard PCR conditions were 94°C for 10 min, followed by 40 cycles at 95°C for 15 seconds, and at 60°C for 1 min. For cytokine mRNA analysis, the gene expression level was calculated by comparison with levels of β-actin mRNA expression in the same sample, using the cycle threshold (Ct) method. The amount of target, was normalized to an endogenous reference (β-actin), relative to a calibrator (uninfected mice), and was given by the ΔCT method using the formula 2^−ΔΔCT^ where CT is the β-actin average, as recommended by the Applied Biosystems user Bulletin #2. Table 4 shows values used for determination of gene expression level of IL-1β by the ΔCT method.

### Detection of degenerated neurons by fluoro-jade B staining.

Fluoro-Jade B (FJB; Chemicon, Temecula, CA, USA) was used to identify degenerated neurons^17^; and for the staining, all tissue sections were deparaffinized in xylene, immersed in a 1% NaOH solution, and rehydrated in distilled water. The sections were immersed in a 0.06% KMnO_4_ solution for 15 min, gently shaken, rinsed in distilled water, and immersed in a 0.0004% FJB staining solution for 20 min. The tissue sections were rinsed in distilled water, rapidly dried at 50°C, immersed in xylene, and coverslipped with *Permount* resin (Fisher Scientific, AL, USA).

### Statistical analysis.

Numbers of nervous tissue cells of different immunophenotypes or producing different cytokines were submitted to a linear model analysis followed by the Student's *t* test. Differences on levels of expression of mRNA of cytokine, TNF-α-producing cells, and apoptotic cells were analyzed by the two-way (analysis of variance) statistical test, followed by Bonferroni's post-test analysis.^18^ Values of *P* < 0.05 were considered as statistically significant. All statistical tests were performed with the GraphPad Prism version 4.0 software (GraphPad Software Inc., San Diego, CA).

## Results

### Animals.

The intraperitoneal inoculation of 10,000 LD_50_ of ROCV in the Balb/C mice resulted in 100% fatality rate and deaths occurred until 9 days a.i. The inoculated animals looked normal 0 through 4 days a.i. and developed signs of mielitis and encephalitis 6 to 9 days a.i (data not shown).

### Immunostaining of ROCV antigens.

**Figure 1.**
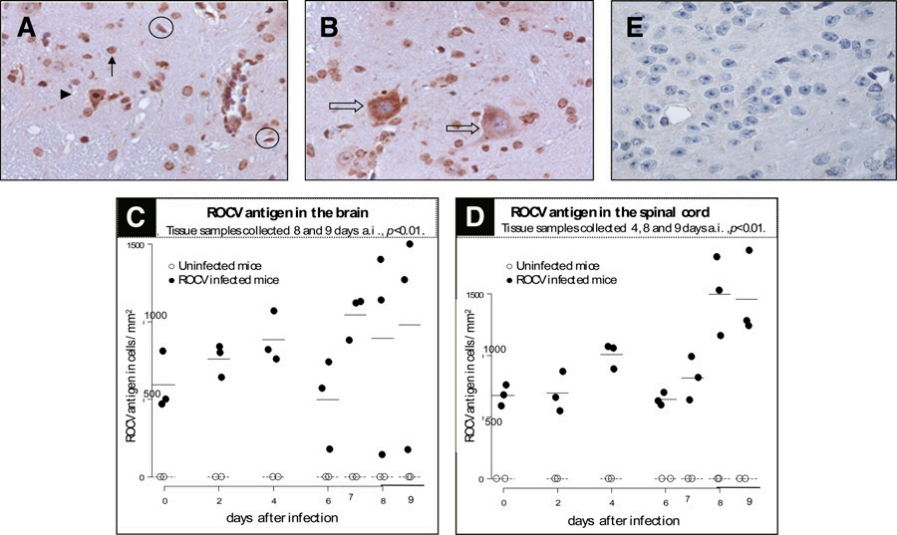
Photomicrographs of nervous tissues of mice infected with Rocio virus (ROCV) harvested 9 days a.i.: (**A**) brain and (**B**) spinal cord. The virus infected, overwhelming the astrocytes (black arrow), neurons (arrow head and open arrow), microglial cells (circles), ependymal cells (at right in **A**), and endothelial cells of capillary (not shown). Immunoperoxidase stains: original magnification: 40×. (**C** and **D)** shows a quantitative analysis, in means of ROCV-positive cells/mm^2^ of nervous tissue (each experiment was repeated three times), in different days after ROCV inoculation, by comparing these results with those from uninfected mice tissues (**E**). Tissue samples were considered statistically significant when obtained with 8 and 9 days a.i., *P* < 0.01.

Immunohistochemical assays of CNS paraffined tissue sections showed ROCV-specific antigens in the brains (Figure 1A) and spinal cords (Figure 1B) collected all days a.i., (0 until 9 days a.i.), in contrast with what was observed in uninfected mice tissue (Figure 1E). The quantification of positive neural cells for ROCV antigen, per square millimeters of volume fraction, in the brain and spinal cord sections, showed increasing means during the infection when compared with the negative control (uninfected mice), and it was specifically significant at Days 4, 8, and 9 (*P* < 0.01; Figure 1C and D).

### Detection of ROCV genome in CNS tissues.

**Figure 2.**
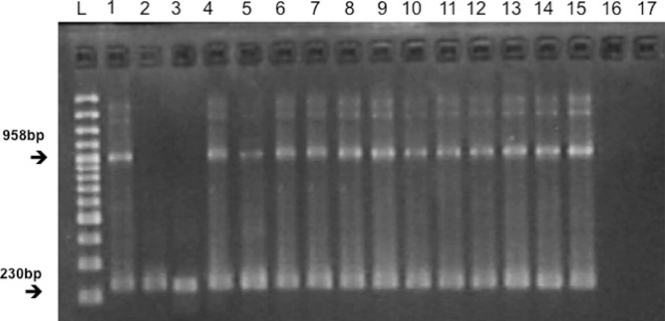
Electrophoresis in agarose gel of the RT-nested-PCR showing *Flavivirus* amplicons of 958 base pair (bp) in lanes 1 and 4–15 and Rocio virus (ROCV)-specific amplicons of 230 bp in lanes 1–15. These samples are from RNA extracts of brain and spinal cord of Balb/C mice infected by ROCV. **L** - Ladder 100 bp; **1** - Positive control (supernatant of macerated brain of mice infected with ROCV; **2** and **3** - infected mice brain and spinal cord collected in the same day of infection—Day 0 after infection (2 h a.i.); **4** and **5** - infected mice brain and spinal cord collected 2 days a.i.; **6** and **7** - infected mice brain and spinal cord collected 4 days a.i.; **8** and **9** - infected mice brain and spinal cord collected 6 days a.i.; **10** and **11** - infected mice brain and spinal cord collected 7 days a.i.; **12** and **13** - infected mice brain and spinal cord collected 8 days a.i.; **14** and **15** - infected mice brain and spinal cord collected 9 days a.i.; **16** and **17-** negative controls, uninfected mice brain and sham-infected mice collected 9 days a.i.

Samples of CNS obtained at Days 0, 2, 4, 6, 7, 8, and 9 a.i., were tested by RT-PCR for detection of *Flavivirus* genus followed by ROCV species-specific nested PCR. ROCV (Flavivirus) amplicons of 958 bp were obtained by RT-PCR from all RNA extracts of cerebral and spinal cord samples except those obtained at Day 0 a.i., as shown in Figure 2. The ROCV-specific amplicons with 230 bp were obtained by nested PCR from samples collected at all times of the experiment, as shown in Figure 2.

### Immunophenotypes of inflammatory cells in CNS tissues.

**Figure 3.**
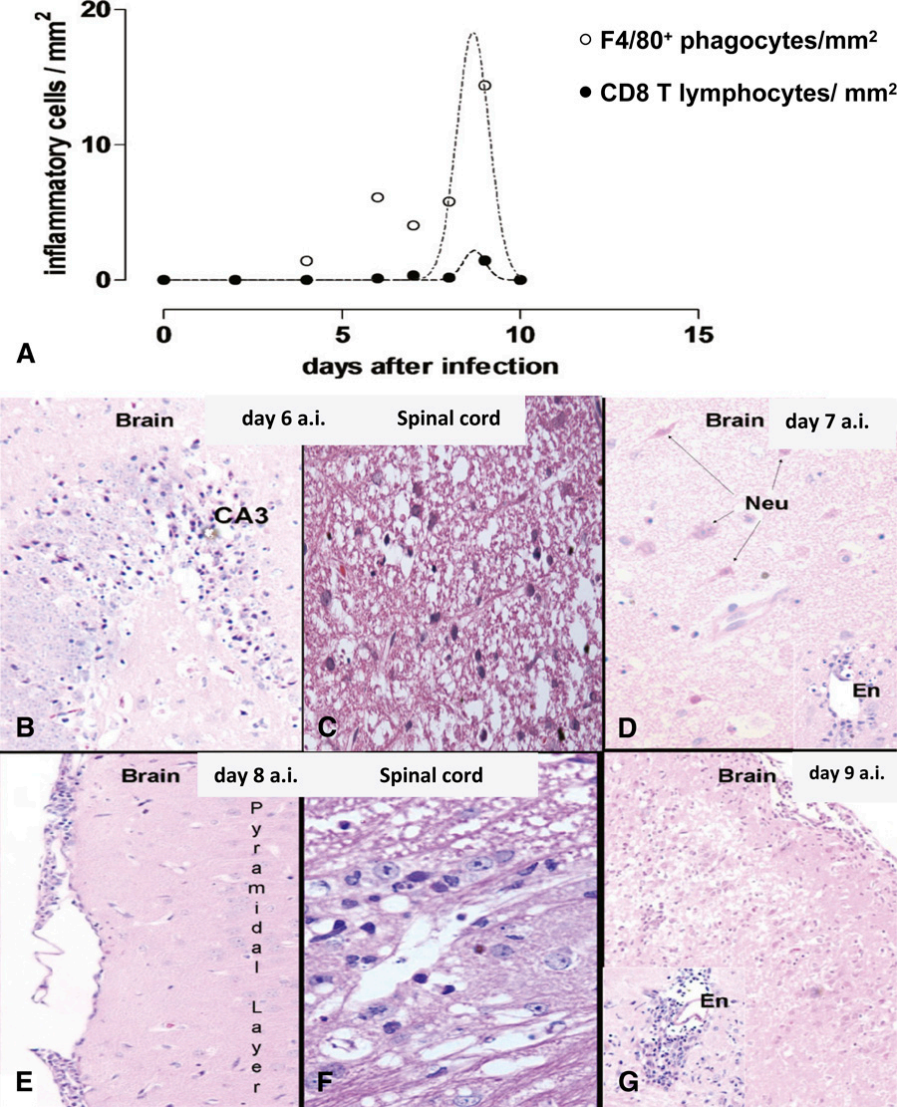
Inflammatory profile of the infection in the brain and spinal cord of Balb/C mice infected by Rocio virus (ROCV). (**A**) Graph showing the kinetics of lymphomononuclear inflammatory cells in central nervous system (CNS) during the infection. (**B**) Photomicrography of hippocampus obtained 6 days a.i., showing a conspicuous neuronal degeneration in the *fascia dentate* (mainly in the CA3 sector). H&E, original magnification: ×20. (**C**) Photomicrography of a spinal cord section obtained 6 days a.i., showing neuropile rarefaction and mild leptomeningitis. H&E, original magnification: ×100. (**D**) Brain section showing degenerated pyramidal neurons in the brain cortex at day 7 a. i. (Neu: pyramidal neuron). H&E, original magnification ×20. Inset, an inflammatory cuff spilled over from capillary is shown (En: endothelium). (**E**) Low power view of a brain cortex obtained at Day 8 a. i., showing a cellular leptomeningitis over the molecular and pyramidal layers. H&E, original magnification: ×10. (**F**) High-power view of a spinal cord obtained at Day 8 a.i., showing interstitial lymphocytes intermingled with astroglial cells. H&E, original magnification: ×40. (**G**) Infected brain obtained 9 days a.i. showing focal encephalitis in the white matter (left lower corner) (En: vascular endothelium). H&E, original magnification: ×10.

**Figure 4.**
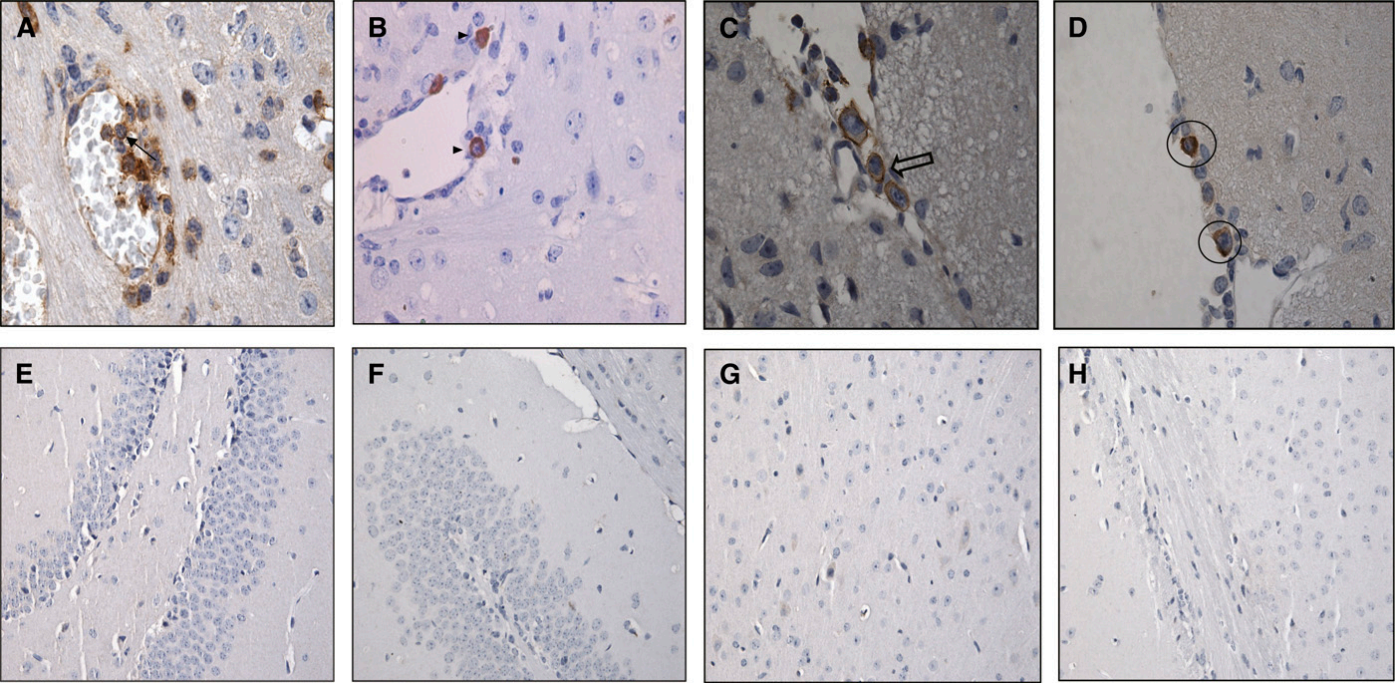
Immunohistochemistry phenotype of inflammatory cells in nervous tissues of mice 9 days a.i. by Rocio virus (ROCV). (**A**) Brain section treated with rat monoclonal antibody (MAb) anti-mouse neutrophil showing neutrophils mixed with glia cells (black arrow) (×100). (**B**) Brain section treated with rat MAb anti-F4/80^+^ monocytic/macrophagic-type cells in the tissue (arrowhead) (×40). (**C**) Brain section treated with rat MAb anti-CD45R showing a leptomeningitis with B lymphocytes and NK cells infiltrate (arrow) (×100). (**D**) Spinal cord section treated with rat MAb anti-CD8 showing a cytotoxic lymphocyte in a venular vessel (circle) (×100). (**E**–**H**) Uninfected brains of mice used as negative controls (×40).

**Figure 5.**
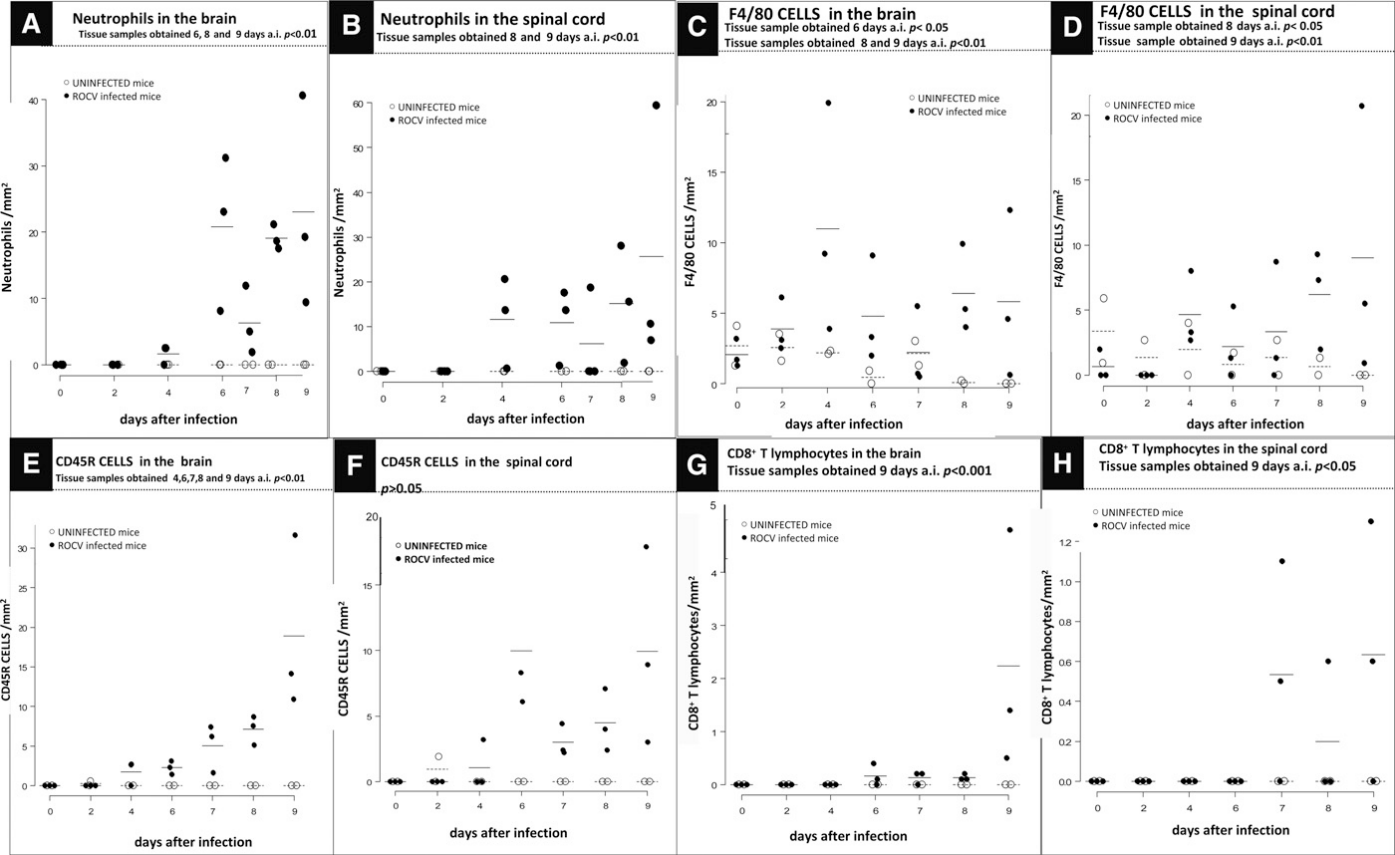
Quantitative analysis of inflammatory cells by mm^2^ of tissue section of brain and spinal cord of mice, at different days a.i. with Rocio virus (ROCV). (**A**–**B**) Neutrophils, (**C**–**D**) F4/80^+^ monocytes/macrophages, (**E**–**F**) CD45R B lymphocytes/NK cells, (**G**–**H**) CD8 T lymphocytes. Inflammatory cells were also counted in nervous tissues of uninfected mice for comparison. (**A**) Neutrophils were found significantly increased in brains obtained 6, 8, and 9 days a.i. (*P* < 0.01). (**B**) Neutrophils were also found significantly increased in spinal cords obtained 6, 8, and 9 days a.i. (*P* < 0.01). (**C**) F4/80^+^ monocytes/macrophages were found significantly increased in brains obtained 6 (*P* < 0.05), 8, and 9 days a.i. (*P* < 0.01). (**D**) In the spinal cord F4/80^+^ monocytes/macrophages were also significantly increased in Days 8 (*P* < 0.05) and 9 (*P* < 0.01) a.i. (**E**) CD45R B lymphocytes/NK cells were significantly increased in brains collected 4, 6, 7, 8, and 9 days a.i. (*P* < 0.01). (**G** and **H**) CD8 T lymphocytes were significantly increased in brains (*P* < 0.01) and spinal cords (*P* < 0.05) collected 9 days a.i.

Brains and spinal cords of ROCV-infected mice harvested 0 to 4 days a.i., did not show pathologic changes (data not shown). Inflammatory cells in nervous tissues of ROCV-infected tissues were seen in significantly higher numbers at Days 4–9 a.i., as compared with the control (uninfected animals) (Figure 3). Lymphomononuclear cells in the inflammatory infiltrates, composed of CD8 T lymphocytes and F4/80^+^ monocytic/macrophage-type cells, showed the highest levels at Days 8 and 9 a.i., in the brains and spinal cords, as shown in Figure 3A. Neuronal degeneration of the hippocampus and pyramidal neurons of the brain cortex was seen associated with cuffs of perivascular inflammation and leptomeningitis, from Day 6 to Day 9 a.i. (Figure 3B, D, E, and G). In the spinal cord, neuropile rarefaction with microglial nodules and mild leptomeningitis were observed from Day 6 to Day 9 a.i. (Figure 3C and F). Inflammatory cells were not observed in nervous tissues harvested from uninfected mice (data not shown). Polimorfonuclear cells were found increased in brains and spinal cords of ROCV-infected Balb/C mice, neutrophils (Figure 4A), escorted by F4/80^+^ monocyte/macrophage-type cells (Figure 4B), CD45R B lymphocytes, *natural killer* cells (Figure 4C), and CD8 T lymphocytes (Figure 4D). All these inflammatory cells in the brain and spinal cord of animals infected by ROCV, at Days 4–9 a.i., were in significant higher numbers (*P* < 0.001, *P* < 0.01, and *P* < 0.05), when compared with these same tissues from uninfected animals (Figure 5). Inflammatory cell infiltrates were not observed in nervous tissues from uninfected animals (Figure 4E–H).

### Neuronal degeneration detected by FJB staining.

**Figure 6.**
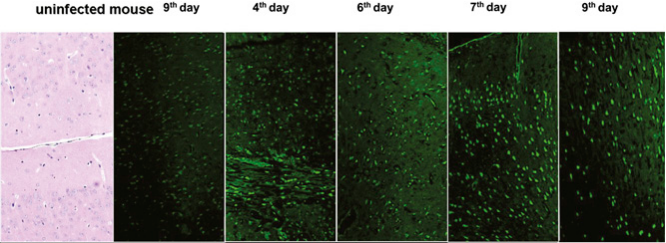
Diffuse neuronal degeneration in fluorescent photomicrographs of sections of brains infected by Rocio virus (ROCV). The sequence of photomicrographs shows an increasing diffuse neuronal degeneration from Day 4 to Day 9 a.i. The left side shows a normal murine brain section as negative control. H&E and Fluoro-Jade B (FJB) stains, original magnifications ×10.

Fluorescent degenerated neurons were found in increasing levels, from Day 4 to Day 9 a.i., in brains and spinal cords from ROCV-infected mice and were not observed in nervous tissues of control animals (uninfected mice) (Figure 6).

### Expression of apoptotic caspase-3.

**Figure 7.**
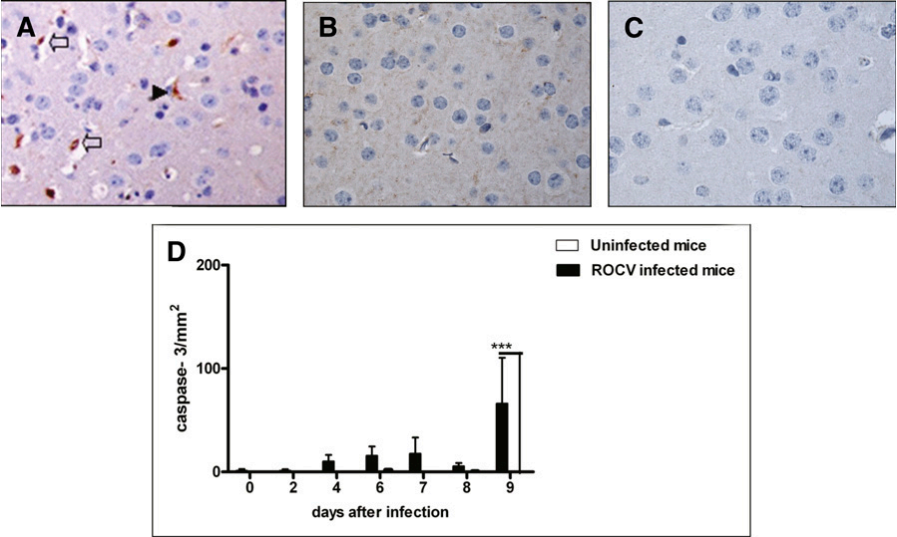
Immunoexpression of caspase-3 in brain sections of mice infected by Rocio virus (ROCV). (**A**) Brain section obtained on 9 days a.i. showing caspase-3-positive glial cells (arrow) and a neuron (head arrow) (×40). (**D**) Graph shows the means of caspase-3-positive cells per volume fraction (means ± SD/mm^2^ of experiments in triplicate) with a significant immunoexpression of caspase-3 on Day 9 a.i. (***P < 0.001). (**B** and **C**) Control brain sections harvested from sham and uninfected mice. (×40).

Cells containing caspase-3 were found in significantly higher levels (***P < 0.001) per squared millimeter of brain and spinal cord of ROCV-infected mice at Day 9 a.i., when compared with the same tissues obtained in previous days of CNS infection (Figure 7). Expression of caspase-3 was not observed in brains or spinal cords from sham and uninfected mice (Figure 7B and C).

### Expression of cytokine mRNAs in nervous tissues.

**Figure 8.**
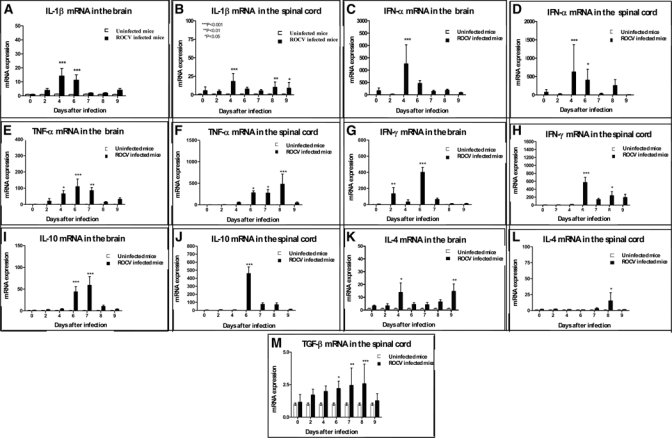
The mRNA of cytokines in brain and spinal cord of mice 0 to 9 days after infection with Rocio virus (ROCV) and in uninfected mice. The levels of IL-1β, IFN-α, TNF-α, IFN-γ, IL-10, IL-4, and TGF-β mRNAs were quantified by real-time polymerase chain reaction (PCR), using the SyberGreen System. The results are presented as the expressions of the target mRNAs with normalization to β-actin. Values (mean ± SD) were obtained from three animals at each time-point, from duplicate measurements, one experiment representative of two. The comparison of levels of cytokine mRNAs in samples of ROCV-infected animals and in samples of uninfected animals showed significant differences: ***P < 0.001; **P < 0.01; *P < 0.05 (two-way analysis of variance with Bonferroni's post-test correction).

The expression of mRNA of Th1-type cytokines in nervous tissues of ROCV-infected mice was significantly increased than in the same tissues of uninfected mice. It was also observed that these expression levels increased following days a.i., as shown in Figure 8. Increased expression of interleukin-1β (IL-1β) mRNA was observed 4 to 6 days a.i. in the brain and 4 days a.i. in spinal cord tissues (*P* < 0.001 for both samples, Figure 8A). These levels remained high 8 (*P* < 0.01) and 9 days a.i., (*P* < 0.05, Figure 8B). The level of IFN-α mRNA was found increased 4 days a.i. in both the brain and spinal cord (*P* < 0.001 for both samples, Figure 8C). Increased expression levels of IFN-α mRNA were also observed 6 days a.i. (*P* < 0.05, Figure 8D). The levels of TNF-α mRNA were found increased in the brain, 4 (*P* < 0.05), 6 (*P* < 0.001), and 7 days a.i. (*P* < 0.01, Figure 8E). In the spinal cord, increased levels of TNF-α mRNA were observed later, 6 (*P* < 0.05), 7 (*P* < 0.05), and 8 days a.i., (*P* < 0.001, Figure 8F). Levels of IFN-γ mRNA were found increased in brain since the second day a.i. (*P* < 0.01) and remained in high levels 6 days a.i., (*P* < 0.001, Figure 8G). In the spinal cord, an increased expression of IFN-γ mRNA was only observed 6 days a.i., (*P* < 0.001), decreasing 8 days a.i., (*P* < 0.05, Figure 8H).

The increased expression of mRNA of Th2-type cytokines occurred later a.i. in nervous tissues of the ROCV-infected mice when compared with that of mRNA of Th1-type cytokines. Increased IL-10 mRNA was detected in brain, 6 (*P* < 0.01) and 7 days a.i. (*P* < 0.001, Figure 8I). The same was observed in the spinal cord where this increased expression had occurred 6 days a.i., (*P* < 0.001, Figure 8J). Increased expression of IL-4 mRNA in the brain was observed 4 (*P* < 0.05) and 9 days a.i. (*P* < 0.01, Figure 8K). Increased expression of IL-4 mRNA in the spinal cord was only observed 8 days a.i., (*P* < 0.05, Figure 8L). The expression of TGF-β mRNA in the spinal cord was found increased 6 (*P* < 0.05), 7 (*P* < 0.01) and 8 days a.i. (*P* < 0.001, Figure 8M). However, increased levels of TGF-β mRNA were not observed in brains of ROCV-infected animals (data not shown).

### Production of cytokines.

**Figure 9.**
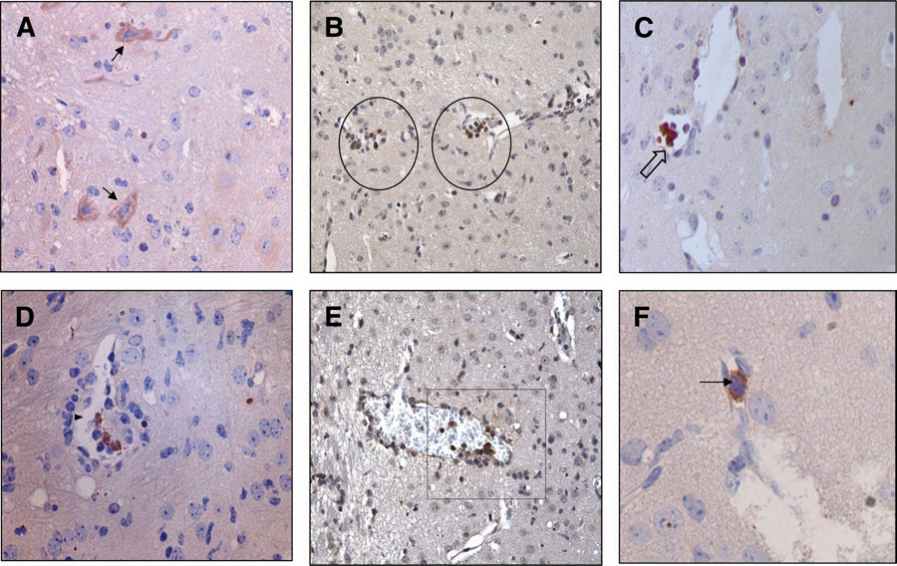
Detection of cytokines in nervous tissue cells after Rocio virus (ROCV) infection. (**A**) Neuron cytoplasmic staining to TNF-α, at Day 9 a.i., in the cortex of the spinal cord (black arrow). (**B**) Neuron cytoplasmic staining to IL-1β, at Day 9 a.i., in the brain cortex (circle). (**C**) Blood vessel leukocytes staining to IFN-α, at Day 7 a.i., in the brain cortex (arrow). (**D**) Perivascular leukocytes staining to IL-10, at Day 9 a.i., in the brain cortex (arrowhead). (**E**) IL-4 stained cells, at Day 8 a.i., in the cortex of the spinal cord (square). (**F**) Perivascular cell stained to TGF-β, 4 days a.i., in the brain cortex (arrow); original magnification, ×40.

**Figure 10.**
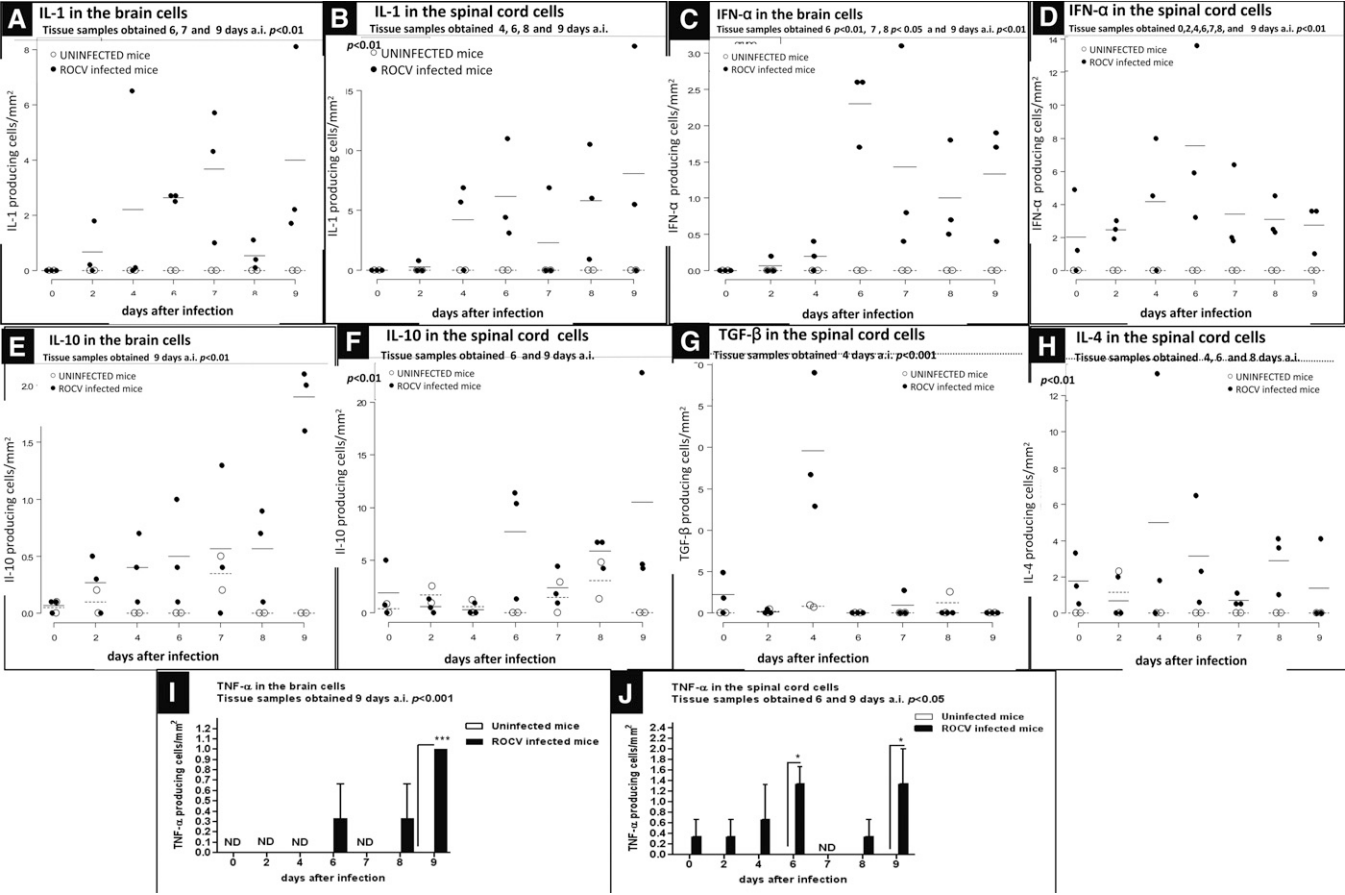
Comparative analysis of the number of cells producing cytokines in the brain and spinal cord (per squared millimeter of tissue preparation) from Rocio virus (ROCV) infected and uninfected mice. Cells producing the following cytokines were analyzed: IL-1β (**A**, **B**), IFN-α (**C**, **D**), IL-10 (**E**, **F**), TGF-β (**G**), IL-4 (**H**), and TNF-α (**I**, **J**). (**A**, **C**, **E**, **F**, **G**, **I**) Brains from ROCV-infected animals showed significantly higher amounts of cells producing cytokines from Days 4 to 9 a.i. (**B**, **D**, **F**, **H**, **J**) Spinal cords from ROCV-infected mice showed significantly higher amounts of cells producing cytokines during the whole period after infection (0 to 9 days). The results are presented as triplicate measurements and also include mean ± SD. ND = not determined.

Cytokines were detected in the brain and in the spinal cord of ROCV-infected mice. TNF-α was observed in the spinal cord markedly stained large and is morphologically consistent with pyramidal neurons (Figure 9A). IL-1β (Figure 9B), IFN-α (Figure 9C), IL-10 (Figure 9D), IL-4 (Figure 9E), and TGF-β (Figure 9F) were also detected in specifically stained cells. The production of IL-1β, IFN-α, IL-10, TGF-β, IL-4 and TNF-α by the nervous tissues of infected animals increased significantly at different times after infection (Figure 10A–J). Low levels of cytokines were observed in nervous tissues of uninfected mice when compared with infected mice (data not shown).

## Discussion

Rocio virus caused a human epidemic of severe encephalitis that lasted from 1973 to 1980 in the Ribeira valley, in the southeastern coast of Brazil. The virus was originally isolated from nervous tissues of a fatal case of encephalitis,^10^ and the disease struck mostly young men after a 7–14-day incubation period. The symptoms were acute fever, headache, anorexia, nausea, vomiting, myalgia, and malaise. Encephalitis signs appeared later, including confusion, reflex disturbances, motor impairment, meningeal irritation, and cerebellar syndrome. Some patients presented with convulsions. Other symptoms included abdominal distension and urinary retention. The disease produced serious sequelae such as visual, olphactory, and auditory disturbances, lack of motor coordination, equilibrium disturbance, swallowing difficulties, incontinence, and defective memory. During this epidemic, 1,021 ROCV cases were reported, ~100 deaths were observed, and more than 200 surviving patients presented with sequelae.^12^,^13^

However, many aspects of ROCV epidemiology remain unknown and the factors responsible for virus appearance and disappearance in the Ribeira valley remain a mystery. The virus was isolated from a wild bird, *Zonothrichia capensis*, and serologic studies suggest that wild birds could be the virus reservoirs. Rocio virus was also isolated from the mosquito *Psorophora ferox*. *Aedes scapularis* is probably another mosquito involved in ROCV transmission. After this outbreak, serologic evidence of ROCV circulation in the Ribeira valley and in other parts of Brazil has been reported and public health authorities are concerned about a return of ROCV outbreaks in Brazil.^19^

In this study, further insights into the immunopathogenesis of the ROCV disease were searched using an experimental model of CNS infection in young adult Balb/C mice. Similar experimental studies have been done to highlight other flavivirus encephalitis, such as those produced by WNV and JRV.^20^,^21^

Rocio virus and other flavivirus related to encephalitis, may reach the nervous tissue after crossing the blood-brain-barrier (BBB)—a functional and structural component of the innate defense that works as an important natural protective factor—throwing into the glial foot-processes strictly apposited on the highly specialized and non-fenestrated endothelial cells of the CNS microvasculature. The physical opposition of the oligodendroglia on the abluminal side of the endothelium, and the secretion of trophic factors by astrocytes, microglial cells, and oligodendrocytes play important roles over the physiology of the BBB.^22^ However, despite the BBB, massive doses (10,000 LD_50_) of ROCV inoculated intraperitoneally allowed the virus to invade the blood stream and to reach CNS of Balb/C mice in < 24 h. Most likely, 2 h a.i., there was a small amount of ROCV in CNS of the infected animals that was only detected by the highly sensitive species-specific nested PCR (Figure 2). It is possible that ROCV has reached the CNS precociously after infection because a high amount of the virus was injected into the animals for the experiments.

The animals developed meningoencephalomyelitis presenting hind limb paralysis, muscle weakness, tremors, and loss of balance (data not shown). In a study on mouse experimental infection by WNV of the sciatic nerve, the virus was transported to the spinal cord producing neuronal injury and acute flaccid paralysis and suggesting that an axonal transport could mediate virus entry into the spinal cord producing myelitis with acute flaccid limb paralysis.^23^ In this study, we observed inflammatory infiltrates and infected neurons at the spinal cord from ROCV-infected mice and was specifically significant at Days 4, 6, 7, 8, and 9 days a.i., and it was followed by acute flaccid limb paralysis of the animals (Figures 1 and 5). However, a significant amount of viral antigens were only observed in neurons, astrocytes, microglia, endothelium, and macrophages/monocytes 4 days a.i. in spinal cords and 8 days in the brains (Figure 1) concomitant to detection of inflammatory immune response. This was different from of a previous report that microglial activation following JEV infection influences the outcome of viral pathogenesis,^24^ in this study, all ROCV-infected mice developed a fatal meningoencephalomyelitis evidenced by hind limb paralysis 6 to 9 days a.i., (data not shown). The disease was anticipated by development of a progressive mixed cell inflammatory infiltrate 4 days a.i. (Figures 4 and 5) coinciding with the presence of the virus in CNS and increasing neutrophils and lymphomononuclear cells. Similar findings were reported with a hamster model after intraperitoneal inoculation of WNV.^25^ The animals developed encephalitic symptoms 6 days a.i. and half of the animals died 7 to 14 days a.i. with histopathologic lesions in hippocampus and brain cortex. A similar inflammatory process in hippocampus as well as in proximal CA3 and distal CA1 regions of CNS was also observed in mice after MVV infection.^26^

The recruitment of inflammatory cells into the nervous tissues of ROCV-infected mice were observed with lymphocytes/NK (CD45R) cells that were detected 4, 6, 8, and 9 days a.i. in the brain (Figure 5). The neutrophils and monocytes/macrophages (F4/80^+^ cells) appeared 6, 8, and 9 days a.i. in the brain and 8 and 9 days a.i. in the spinal cord (Figures 4 and 5). CD8^+^ T lymphocytes were detected in the brain and spinal cord, 9 days a.i. (Figures 4 and 5). Inflammatory cells in CNS produced cytokines that were detected based on specific RNAm amplification and cytokine staining in the cells. The production of IFN-α in the CNS was probably immediate because it was found significantly increased < 24 h after ROCV infection in the spinal cord and remained at high levels during the whole infection period (Figures 9 and 10). Likewise, RNAm of IFN-α was detected in the brain 4 days after ROCV infection (Figure 8), probably produced by monocytes/macrophages. IFN-α induces innate antiviral activity and increases the cytolytic activity of NK cells.^27^ The IFN-α action can promote survival of cells such as astrocytes, neutrophils,^28^ and also can preserve neurons and glial cells by enhancing virus clearance.^29^ The mRNA of Th1 cytokines such as IFN-γ and IL-1β was first detected in mouse nervous tissues 2 and 4 days after ROCV infection (Figure 8). The IFN-γ and IL-1β, both induce production of nitric oxide and peroxynitrite affecting viral replication.^24^ The importance of IFN-γ as a defense tool in flavivirus infection was demonstrated in a study with IFN-γ (or its receptor) knockout C57Bl/6 mice. These animals, when infected by WNV, presented a higher viremia, with intense viral replication in lymphoid tissues and a higher mortality compared with normal animals.^30^ In this study, TNF-α RNAm was first detected 4 days a.i. (Figure 8) and this cytokine was only significantly increased after 2 days (Figure 10).

The IL-4, IL-10, and TGF-β mRNA, both Th2-type cytokines, were found in nervous tissues 4 days after ROCV infection (Figures 8 and 9). The IL-10, another Th2-type cytokine, was detected only 6 days a.i. The Th2-type cytokines within CNS were correlated with the onset of meningoencephalomyelitis in the infected mice (Figure 8). These cytokines could be involved with homeostatic control of the inflammatory response, acting as a negative feedback for proinflammatory cytokines such as IL-1β and TNF-α.^27^ The expression of Th2-type cytokines could have protected the infected mice by down-regulating the inflammatory response in their nervous tissues. To reduce inflammation is important for the protection of nervous tissues that are fragile and highly sensitive to the hypoxia produced by edema.^31^ Other studies have observed a progressive decline of IL-10 levels after JEV infection in the CNS, and it was inversely proportional to the increase of proinflammatory cytokine levels, worsening prognosis of the infection.^32^

The Th1- and Th2-type cytokines present in nervous tissues of the ROCV-infected animals suggests that this infection induces a balance of cytokines to accomplish a suitable immune response.^27^ However, even this mixed Th1- and Th2-type immune response was not able to control the ROCV disease of the mice. All the infected animals became sick and died.

Six days after ROCV infection, besides the inflammatory process in CNS, severe tissular lesions were detected. Neuron degeneration was observed in the hippocampus, dentate gyrus, and CA3 region by FJB staining (Figure 6). Fluoro-Jade, an anionic fluorochrome capable of selectively staining degenerated neurons, was able to show the extensive destruction of these cells during ROCV infection.^17^

Degeneration and death of neurons, most likely occurred as a consequence of ROCV direct infection and apoptosis induced by cytokines produced by glial and macrophage activated cells. Apoptotic cells producing caspase 3, including neurons, lymphomononuclear, and endothelia cells, were significantly increased in the later phase of ROCV infection at different regions of the CNS (Figure 7). Apoptosis of astrocytes was not observed in this study, corroborating a previous report on murine astrocytes that were infected *in vitro* by WNV. These cells developed a persistent infection and survived, whereas neurons were destroyed by the virus.^33^

As part of the planning for this work, the analysis of brain and spinal cord tissues was performed separately to detect particular differences on disease pathology at these two different places. However, with exception of a slight delay on appearing and a lower production of some cytokines by spinal cord, pathologic differences were not evident.

In short, using this mouse experimental model of meningoencephalomyelitis, it was possible to follow-up and to study the complete cycle of infection by ROCV in the CNS, including inflammatory changes induced by cytokines and the final irreversible damage of the nervous tissues. These observations can help to understand the pathogenesis and physiopathology of the human meningoencephalomyelitis by ROCV and other flaviviruses.

## Figures and Tables

**Table 1:** Antibodies used for immunohistochemistry analysis of mouse nervous tissues

Primary and secondary antibodies	Dilution	Source	Clone or lot number
PAb goat anti-rat IFN-γ	1:10	R&D Systems, MN	AHX01
PAb goat anti-mouse TNF-α	1:10	R&D Systems, MN	NQ09
PAb goat anti-mouse IL-1β	1:6 R&D Systems, MN	NP10	
PAb goat anti-mouse IL-10	1:10 R&D Systems, MN	BPZ02	
PAb rabbit anti-mouse IL-4	1:10	Serotec, Oxford, UK	AAM36
PAb rabbit anti-mouse IFN-α	1:100	PBL Biomedical, NJ	32100-1
PAb goat anti-human TGF-β	1:20	R&D Systems, MN	EF01
MAb rat anti-mouse CD8	1:40	Serotec, Oxford, UK	KT15
MAb rat anti-mouse CD45R	1:200	Serotec, Oxford, UK	RA3-6B2
MAb rat anti-mouse F4/80	1:200	Serotec, Oxford, UK	CI:A3-1
MAb rat anti-mouse neutrophil	1:150	Serotec, Oxford, UK	7/4
MAb mouse anti-caspase-3	1:200	Novocastra, Newcastle, USA	JHM62
MAb mouse anti-E protein			
of flavivirus (4G2)	1:80	Supplied by Dr. Peter Mason	
PAb rabbit anti-goat IgG	1:200	Bethil Laboratory, TX	
PAb goat anti-rat IgG	1:200	Zymed Laboratory, CA	
PAb goat anti-rabbit IgG	1:200	Bethil Laboratory, TX	
PAb horse anti-mouse IgG	1:200	Novocastra, Newcastle, USA	
PAb rabbit anti-mouse IgG	1:200	Dako, NY, USA	

**Table 2:** Primer sequences used for the reverse transcription-polymerase chain reaction (RT-PCR) for flavivirus genome detection followed by a nested PCR for Rocio virus (ROCV)

Name	Sense and complementary primers
Flavivirus-S	TCAAGGAACTTCACACATGAGATGTACT
Flavivirus-C	GTGTCCCATCCTGCTGTGTCATCAGCATACA
Flavivirus-S	TCAAGGAACTTCACACATGAGATGTACT
ROCV-C	TCACTCTTCAGCCTTTCG

**Table 3:** Primers, including their melting temperatures and amplicon size of cytokine messenger RNAs, as explained by the manufacturer **(**Bionner, Califórnia, USA)

Target	Sense and anti-sense sequences	*t*M (°C)	bp
IL-1β	CAACCAACAAGTGATATTCTCCATG	81	152
	GATCCACACTCTCCAGCTGCA		
TNF-α	CATCTTCTCAAAATTCGAGTGACAA	86	175
	TGGGAGTAGACAAGGTACAACCC		
IFN-γ	TCAAGTGGCATAGATGTGGAAGAA	78	92
	TGGCTCTGCAGGATTTTCATG		
IL-4	CTGACGGCACAGAGCTATTGA	82	251
	TATGCGAAGCACCTTGGAAGC		
IFN-α	ACTGGCCAACCTGCTCTCTA	83	172
	CTTCTTGATCTGCTGGGCA		
IL-10	GGTTGCCAAGCCTTATCGGA	82	191
	ACCTGCTCCACTGCCTTGCT		
TGF-β1	TGACGTCACTGGAGTTGTACGG	83	170
	GGTTCATGTCATGGATGGTGC		
β-actina	AGAGGGAAATCGTGCGTGAC	82	138
	CAATAGTGATGACCTGGCCGT		

Mt = melting temperature; bp = base pairs of amplicon size; IFN = interferon; IL = interleukin; TNF = tumor necrosis factor; TGF = tumor growth factor.

**Table 4:** Values used for determination of gene expression level of IL-1β by ΔCT method

β-actin average Ct	Target gene average Ct	ΔCt = β-actin–target gene	ΔΔCT = ΔCt infected animal–control animal ΔCT
17, 72	28, 49	10, 67	−3, 05
